# Effects of omega-6/3 and omega-9/6 nutraceuticals on pain and fertility in peritoneal endometriosis in rats^1^

**DOI:** 10.1590/s0102-865020190040000005

**Published:** 2019-05-06

**Authors:** Francisco Edson Ximenes Gomes Pereira, Francisco das Chagas Medeiros, Hermano Alexandre Lima Rocha, Karine Saraiva da Silva

**Affiliations:** I Fellow PhD degree, Postgraduate Program in Medical and Surgical Sciences, Department of Surgery, Universidade Federal do Ceará (UFC), Fortaleza-Ce, Brazil. Conception of the study, technical procedures, acquisition of the data, manuscript writing.; II PhD, Associate Professor, Department of Maternal and Child Health, UFC, Fortaleza-Ce, Brazil. Conception, design, intellectual and scientific content of the study; critical revision; final approval.; III PhD, Associate Professor, Department of Medical Clinic, UFC, Fortaleza-Ce, Brazil. Statistical analysis.; IV Graduate student, UFC, Fortaleza-Ce, Brazil. Acquisition of data.

**Keywords:** Endometriosis, Dietary Supplements, Fertility, Pain, Fatty Acids, Omega-3, Rats

## Abstract

**Purpose:**

To evaluate the effects of the nutraceuticals omega-6/3 and omega-9/6 on endometriosis-associated infertility and pain.

**Methods:**

Controlled experimental study, with each group composed of eight female rats. Fertility groups: sham-operated control (0.9% saline solution); control with endometriosis (0.9% saline); omega-6/3 (1.2 g/kg/day); omega-9/6 (1.2 g/kg/day); and meloxicam (0.8 mg/kg/day). Pain groups: sham-operated control (0.9% saline); control with endometriosis (0.9% saline); omega-6/3 (1.2 g/kg/day); omega-9/6 (1.2 g/kg/day); medroxyprogesterone acetate (5 mg/kg/every 3 days); and meloxicam (0.8 mg/kg/day). Peritoneal endometriosis was surgically induced. Pain was evaluated with the writhing test. Fertility was evaluated by counting the number of embryos in the left hemi-uterus.

**Results:**

The mean number of writhings was as follows: sham-operated, 11.1 ± 2.9; control with endometriosis, 49.3 ± 4.4; omega-6/3, 31.5 ± 2.7; omega-9/6, 34.1 ± 4.5; medroxyprogesterone acetate, 2.1 ± 0.8; meloxicam, 1 ± 0.3. There was a significant difference between both controls and all drugs used for treatment. Regarding fertility, the mean values were as follows: sham-operated, 6.8 ± 0.6; control with endometriosis, 4.2 ± 0.7; omega-6/3, 4.7 ± 1; omega-9/6, 3.8 ± 0.9; and meloxicam, 1.8 ± 0.9.

**Conclusions:**

The omega-6/3 and omega-9/6 nutraceuticals decreased pain compared to the controls. There was no improvement in fertility in any of the tested groups.

## Introduction

 Endometriosis is defined as the presence of endometrial tissue (glands and stroma) outside the uterine cavity^1^. It is a common disease among women of reproductive age and is clinically characterized by dysmenorrhea, infertility and dyspareunia^2^.

 Rapid changes in the human diet, especially in the last 100 years, have been potent promoters of chronic diseases such as atherosclerosis, essential hypertension, obesity, diabetes and many types of cancer^3^. In the Western diet, the ratio of omega-6/omega-3 fatty acids ranges from 10:1 to 30:1, which is very different from the ratio of 1:1 to 2:1 in the diets of prehistoric populations^4^. The optimal dose or ratio of omega-6/omega-3 ranges from 1:1 to 1:4 depending on the disease considered^5^. The importance of the omega-6/omega-3 ratio was demonstrated in an apolipoprotein E (ApoE)-deficient mouse model that also expressed the fat-1 gene of *Caenorhabditis elegans*. The fat-1 transgenic mouse metabolizes omega-6 to omega-3 through an omega-3 desaturase enzyme, resulting in an approximate 1:1 omega-6/omega-3 ratio. After feeding with a high-fat diet for fourteen weeks, the ApoE^-/-^ fat-1 transgenic mouse was found to have fewer atherosclerotic lesions than the ApoE^-/-^ mouse^6^. Omega-6 fatty acids account for most polyunsaturated fatty acids in diets, especially the Western diet. When the diet is supplemented with omega-3 fatty acids, omega-6 fatty acids are partially replaced in virtually all cell membranes^7^. A study published in the 1980s suggesting the importance of consuming omega-3 polyunsaturated fatty acids was based on epidemiological observations of the low incidence of autoimmune and inflammatory disorders in a Greenland Eskimo population compared with that in groups living in Denmark^8^. With a Mediterranean diet providing a linoleic acid (LA):alpha-linolenic acid (ALA) ratio of 4:1, increased incorporation of ALA into cell membranes was observed. The 4:1 ratio of LA:ALA led to a 70% reduction in total mortality at the end of two years^9^. Omega-3 fatty acids affect interleukin metabolism by decreasing interleukin-1β (IL-1β) and interleukin-6 (IL-6) levels^5^. Interleukin-6 may stimulate the synthesis of all acute-phase proteins involved in the inflammatory response, such as C-reactive protein, serum amyloid A, fibrinogen, α1-chymotrypsin and haptaglobin^10^. Omega-3 fatty acids suppress the ability of monocytes to synthesize interleukin-1 (IL-1) and tumor necrosis factor (TNF) in healthy volunteers^7^. Omega-3 fatty acids inhibit the production of nuclear factor-kappa beta (NF-kB), which is a transcription factor for a large number of cytokines such as tumor necrosis factor alpha (TNF-α) and interleukins^11^.

 Inflammation is one of the major mechanisms underlying visceral pain, and endometriosis is an inflammatory disease that triggers an inflammatory response^12^. Endometriosis occurs most frequently in the pelvic viscera and the peritoneum of the pelvic viscera; thus, endometriosis-associated pain is usually of a visceral origin^13^. The mechanism of endometriosis-associated infertility is not well understood, although endometriosis is known to impair ovarian and tubal function as well as reduce uterine receptivity. Abnormal folliculogenesis, high oxidative stress, and immunological changes may also contribute to decreased fertility. Endometriosis is associated with infertility and a low pregnancy rate due to poor quality of oocytes and embryos^14^. Endometriosis is associated with inflammatory changes in the intrafollicular microenvironment^15^. The levels of inflammatory cytokines, such as interleukin-6 (IL-6), interleukin-1β (IL-1β) and tumor necrosis factor alpha (TNF-α), are increased in the follicular fluid of patients with endometriosis. These inflammatory cytokines can activate apoptosis^14^. Oxidative stress is initiated by inflammatory cells, with cellular debris acting as a substrate^16^. Some studies have shown that nutritional status and dietary intake may play a role in reproductive health^17^. The Mediterranean diet (rich in vegetable oils, vegetables, fish and legumes) was positively associated with folate in red blood cells and vitamin B6 in blood and follicular fluid, leading to a 40% increase in the likelihood of becoming pregnant^18^. Hammiche *et al.* ^19^ showed that women with high omega-3 intake had a reduced number of follicles and an improved embryo morphology after ovarian stimulation. These results suggest that mild ovarian stimulation results in few follicles, allowing only healthy follicles and oocytes to develop into competent embryos. Therefore, fish consumption at least twice a week is recommended for women in their reproductive years, especially those undergoing in vitro fertilization. Jungheim *et al.* ^20^ reported that the increase in the ratio between LA and ALA was associated with a significant increase in the likelihood of becoming pregnant and had a positive correlation with embryo implantation.

 In the literature, several interpretations of the terms functional food and nutraceutical are available.

 The term “nutraceutical” was derived from “nutrition” and “pharmaceutical” in 1989 by Stephen DeFelice, founder and president of the Foundation for Innovation in Medicine. According to DeFelice, a nutraceutical can be defined as “a food or part of a food, such as a dietary supplement, that has a medical or health benefit, including the prevention and treatment of disease”^21^.

 The most recent definition of functional foods was proposed at the 17th International Conference organized by the United States Department of Agriculture (USDA) and Agricultural Research Service (ARS) in 2014: “Natural or processed foods that contain known or unknown biologically-active compounds; which provide a clinically proven and documented health benefit for the prevention, management or treatment of chronic disease”^22^.

 Nutraceuticals can be categorized as probiotics, prebiotics, dietary fiber, antioxidant vitamins, polyphenols, spices (seasoning) and polyunsaturated fatty acids^23^.

 The aim of this study was to evaluate the effect of omega-6/3 and omega-9/6 nutraceuticals on endometriosis-associated pain and infertility.

## Methods

 Wistar albino rats (*Rattus norvegicus*) were used. The animals weighed approximately 150-170 g and were up to 3 months of age. Each experimental group consisted of 8 female rats, totaling 48 rats, which were fed standard feed and water *ad libitum*. Light/dark cycles were alternated every 12 hours. This study was approved by the Animal Research Ethics Committee, School of Medicine, Universidade Federal do Ceará, protocol 88/2014 on 10/30/2014.

 Peritoneal endometriosis was surgically induced based on the model developed by Vernon *et al.* ^24^. To induce pain, we used the abdominal contortion (writhings) test. Acetic acid at 0.6% 10 mL/kg (compounded by Farmafórmula Ltda^®^) was injected intraperitoneally, and then the rats were individually placed in cages, allowing an optimal number of abdominal contortions in 20 minutes, with counting initiated 5 minutes after injecting the acetic acid. An abdominal contortion (writhe) was defined as a sequence of arching of the back, pelvic rotation and hind limb extension. The mean number of abdominal contortions in each group was determined for comparison with the other groups. The control without endometriosis group (sham-operated) and the control with endometriosis group received 0.9% saline soution^25^. Four treatment groups were established for pain evaluation: the low omega-6/3 ratio (1.4:1) group (1.2 g/kg/day orally by gavage); the high omega-9/6 ratio (3.7:1) group (1.2 g/kg/day via orally by gavage); the medroxyprogesterone acetate group (5 mg/kg/3 days subcutaneously (sc)); and the meloxicam group (0.8 mg/kg/day orally by gavage). The omega fatty acids mixture was compounded by a specialized nutritionist. The omega-3 used was manufactured by OH2 Nutrition Ind. Suplementos Alimentares^®^ and distributed by Farmafórmula Ltda^®^ (DHA/EPA 1 cap.= 1,000 mg); the omega-6 (soybean oil) and omega-9 (olive oil) were purchased in a supermarket. The medroxyprogesterone acetate used was Depo-Provera^®^ (Laboratórios Pfizer Ltda) and the meloxicam was manufactured by Eurofarma Laboratórios SA. All rats were treated for 9 days starting on day five after implantation, and they were sacrificed on day fourteen after implantation.

**Figure 1:**
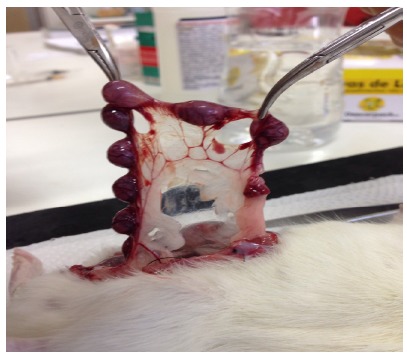
Embryos developing in the left hemi-uterus of the rat. Each nodule represents an embryo developing in the left hemi-uterus of the rat. The number of embryos was used to evaluate fertility.

 To analyze the effect of surgically induced endometriosis on fertility, the females from the control without endometriosis group (sham-operated), control with endometriosis group and all treatment groups were mated with males with proven fertility. Each mating cage had three males for each female. The reproductive cyclicity of all female rats was examined by daily vaginal smears, and the day of conception (day 1) was determined by the presence of spermatozoa in vaginal lavage fluid. Pregnant rats were then sacrificed by anesthetic overdose on the tenth day of pregnancy, and the number of embryos implanted in the left uterine horn were counted (Fig. 1). A control group without endometriosis and a control group with endometriosis were used. The treatment groups were omega-6/3 low ratio 1.4:1 (1.2 g/kg/day by oral gavage), omega-9/6 high ratio 3.7:1 (1.2 g/kg/day by oral gavage) and meloxicam (0.8 mg/kg/day by oral gavage) (Eurofarma Laboratórios S. A.). All rats were treated for 9 days, starting on day five after implantation. One-way ANOVA or Brown-Forsythe statistical tests were used according to the results of Levene’s homogeneity test.

## Results

**Figure 2:**
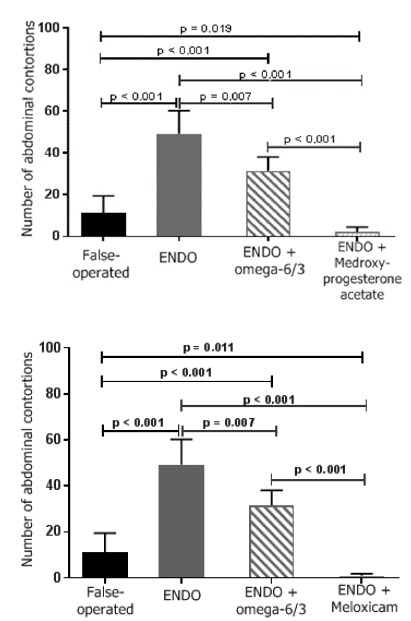
Effect of treatment with ω-6/3, medroxyprogesterone acetate and meloxicam on abdominal contortions (writhings) induced by acetic acid in sham-operated rats and rats with peritoneal endometriosis.

Legend: ENDO: group with endometriosis; groups of rats subjected to the writhings test (0.6% acetic acid; 10 mL/kg ip). Sham-operated group: not treated; ENDO group: with endometriosis and not treated; and ENDO + ω-9/6 group (1.2 g/kg/day; vo), ENDO + medroxyprogesterone acetate group (5 mg/kg/3 days sc) and ENDO + meloxicam group (0.8 mg/kg/day). The Kolmogorov-Smirnov test indicated an approximately normal distribution for all variables. Due to the high heterogeneity of variances, two parametric tests were performed, according to Levene’s homogeneity test: one-way ANOVA or the Brown-Forsythe test.

**Figure 3:**
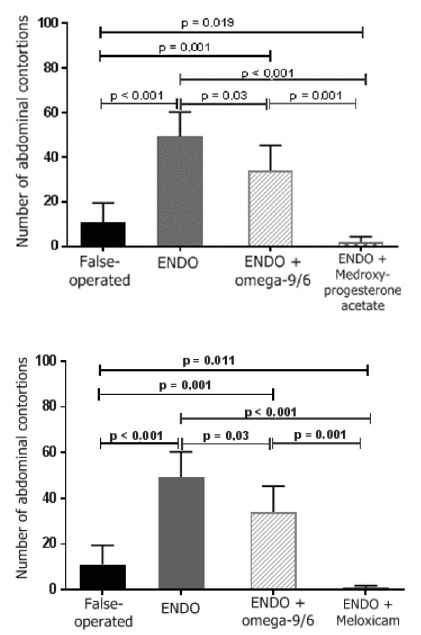
Effect of treatment with ω-9/6, medroxyprogesterone acetate and meloxicam on abdominal contortions (writhings) induced by acetic acid in sham-operated rats and rats with peritoneal endometriosis.

Legend: ENDO: group with endometriosis; groups of rats subjected to the writhings test (0.6% acetic acid; 10 mL/kg ip). Sham-operated group: not treated; ENDO group: with endometriosis and not treated; and ENDO + ω-9/6 group (1.2 g/kg/day; vo), ENDO + medroxyprogesterone acetate group (5 mg/kg/3 days sc) and ENDO + meloxicam group (0.8 mg/kg/day). The Kolmogorov-Smirnov test indicated an approximately normal distribution for all variables. Due to the high heterogeneity of variances, two parametric tests were performed, according to Levene’s homogeneity test: one-way ANOVA or the Brown-Forsythe test.

 Pain was assessed by the arithmetic mean ± standard error of the mean (SEM) of the number of abdominal contortions of the female rats in the control and treatment groups. The mean number of abdominal contortions were as follows: controls without endometriosis (sham-operated) 11.1 ± 2.9; controls with endometriosis, 49.3 ± 4.4; rats treated with omega-6/3 1.2 g/kg/day, 31.5 ± 2.7; rats treated with omega-9/6 1.2 g/kg/day, 34.1 ± 4.5; rats treated with medroxyprogesterone acetate 5 mg/kg/every 3 days, 2.1 ± 0.8; and rats treated with meloxicam 0.8 mg/kg/day, 1 ± 0.3. A significant difference was observed between the controls without endometriosis (sham-operated) and the controls with endometriosis and between both control groups and all drug treatment groups (Figs. 2 and 3). No difference was found between the omega fatty acid treatment groups. 

**Figure 4:**
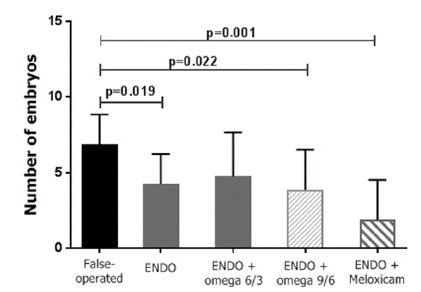
Effect of treatment with ω-6/3, ω-9/6, medroxyprogesterone acetate and meloxicam on the fertility of sham-operated rats and rats with peritoneal endometriosis.

Legend: ENDO: group with endometriosis; evaluation of fertility in sham-operated rats: not treated; ENDO group: not treated; ENDO + ω-6/3 group (1.2 g/kg/day vo); ENDO + ω-9/6 group (1.2 g/kg/day vo); ENDO + meloxicam group (0.8 mg/kg/day vo). The Kolmogorov-Smirnov test indicated an approximately normal distribution for all variables. Due to the high heterogeneity of variances, two parametric tests were performed, according to Levene’s homogeneity test: one-way ANOVA or the Brown-Forsythe test.

 Fertility was assessed by the arithmetic mean ± standard error of the mean of the number of embryos developing in the left hemi-uterus of the female rat. A control group without endometriosis (sham-operated) and a control group with endometriosis were used. The mean values of the control and treatment groups were as follows: controls without endometriosis, 6.8 ± 0.6; controls with endometriosis (sham-operated), 4.2 ± 0.7; rats treated with omega-6/3, 4.7 ± 1; rats treated with omega-9/6, 3.8 ± 0.9; and rats treated with meloxicam, 1.8 ± 0.9. A difference was found between the controls (sham-operated) and the controls with endometriosis. A difference was found between the controls (sham-operated) and the groups treated with omega-9/6 and meloxicam. No difference was found between the controls with endometriosis and the treatment groups or between the different drug treatment groups (Fig. 4).

## Discussion

 As observed, both omega-6/3 and omega-9/6 did not perform better than medroxyprogesterone acetate and meloxicam, but they had an analgesic effect, probably due to anti-inflammatory^5^ ^,^ ^26^ and antioxidant effects^27^. The reason why they did not perform better than medroxyprogesterone acetate and meloxicam may be because in this study, the polyunsaturated fatty acids were used as nutraceutical treatment, whereas in most studies, they were used as a supplement. Even when used as a treatment, the results of this study are consistent with those of several studies in the literature. Mesquita *et al*.^28^ supplemented healthy female rats with omega-3 at a dose of 200 mg/kg/day for 20 days and compared this treatment with tenoxicam 1 mg/kg/day and saline, using the formalin test to assess pain. A difference was identified between the omega-3 group and the saline group, but no difference was found compared to tenoxicam, with tenoxicam exhibiting a greater tendency to decrease pain; conversely, when meloxicam was used in this study, a large difference was observed compared to omega-6/3 and omega-9/6. Zafari *et al*.^29^ compared the pain intensity associated with primary dysmenorrhea between groups treated with ibuprofen 400 mg/day and fish oil 1000 mg/day taken during the menstrual period and found differences between them, with the fish oil performing better. That study was conducted with women, unlike the present study, which used meloxicam in rats. However, the finding that the effect of fish oil was superior to ibuprofen is interesting. Zillioux *et al*.^30^ reported decreased allodynia in rats after surgically inducing peripheral nerve injury by comparing groups treated with omega-3 (DHA and EPA), soybean oil and omega-3 combined with aspirin and a sham-operated group. No difference was found between the omega-3 group and the group treated with omega-3 combined with aspirin, but a difference was observed between the omega-3 group and the other groups. This experiment strengthens the result obtained in the writhings test of rats treated with a mixture of omega-6/3 and omega-9/6. Galán-Arriero *et al*.^31^ reported that administration of oleic acid (omega-9) in combination with albumin promotes recovery of motor function and reduces spasticity after spinal cord injury, in addition to promoting antinociception and anxiolytic effects after peripheral and central nerve injury. Only omega-9 was used in that study, but it was sufficient to show the viability of using polyunsaturated fatty acids for the treatment of conditions involving an inflammatory process, which supports treatment that involves the omega-9/6 mixture.

 Regarding fertility, the results of this experiment diverge from those in the literature because studies using omega-3 polyunsaturated fatty acids as a supplement improve fertility. The results in this work probably differ from those in the literature because polyunsaturated fatty acids were used as a nutraceutical treatment since omega-6/3 has an anti-inflammatory effect^5^ ^,^ ^26^ and omega-9/6 has an antioxidant effect^27^, which secondarily acts as an anti-inflammatory by decreasing oxidative stress, thus reducing the pro-inflammatory cytokines induced by such stress. Therefore, these nutraceuticals may have decreased the inflammatory reaction, which usually occurs at the blastocyst implantation site. Meloxicam, which is also an anti-inflammatory and did not yield results differing from those of the controls with endometriosis, may also act through this mechanism; that is, by reducing inflammation at the blastocyst implantation site, implantation would be impaired due to absence of the inflammatory reaction necessary for implantation to occur.

 Cyclooxygenases (COX) 1 and 2 are responsible for the production of prostaglandins. In the endometrium, COX-1 production decreases in response to progesterone and 17β-estradiol, and the level of COX-1 decreases in the middle luteal phase. COX-2 production, which is not affected by steroid hormones, is restricted to the implantation site and depends on the presence of a blastocyst that is ready to implant. Interleukin-1 in human embryo culture medium induces the expression of the COX-2 gene in endometrial stromal cells^32^. The omega-6/3 and omega-9/6 used as treatments in this study potentially inhibit COX-2 depending on the presence of the blastocyst.

 Eskew *et al.* ^33^ did not find any correlation between the omega-3 index in the erythrocytes of women subjected to in vitro fertilization and the number of recovered oocytes, the mean number of fertilized oocytes or the fertilization rate. That is, fertility was not influenced by the omega-3 index. That study found similar results to the present study, although it was a human study. Chiu *et al.* ^34^ found that higher serum omega-3 levels acquired through diet were associated with a higher probability of clinical pregnancies and live birth in women undergoing assisted reproductive therapy. That study was conducted with women, and the omega-3 was offered through the diet, unlike the present experiment in which omega-3 was used as the treatment, and the results obtained are discordant^34^. 

 Using omega-6 and omega-3 supplementation in an investigation of uterine phospholipid fatty acid composition, Fattahi *et al*.^35^ found that on the first day of pregnancy, the levels of arachidonic acid and total omega-6 fatty acids were much higher than the levels of linolenic acid and total omega-3 fatty acids. The total omega-6 fatty acids level was also positively correlated with the embryo implantation rate in the control group and in the omega-6 supplemented group on the fifth day of pregnancy^35^. The findings of these authors reinforce the hypothesis raised in the present study, suggesting that the nutraceutical treatment could have blocked the inflammatory reaction at the time of blastocyst implantation, thus impairing fertility. Nassan *et al.* ^36^ compared the intake of meat and other protein-rich foods on assisted fertilization treatment and observed that women who had a higher pretreatment intake of fish had a higher probability of live birth^36^. The results of these authors are in accordance with the literature and thus contrary to the results of the present study, which used omega fatty acids as nutraceutical and did not obtain good results regarding fertility.

## Conclusions

 The omega-6/3 and omega-9/6 nutraceuticals decreased pain to a lesser extent than medroxyprogesterone acetate and meloxicam, but the reduction was significant relative to the controls. Regarding fertility, the results were inconsistent with the literature, probably because the nutraceuticals were used as a treatment rather than a supplement, affecting the inflammatory reaction at the blastocyst implantation site. Further studies are recommended to confirm the results obtained in this article.
